# Utility of oral fluid samples for hepatitis B antibody detection in real life conditions

**DOI:** 10.1186/s12879-019-4183-0

**Published:** 2019-07-17

**Authors:** Helena Medina Cruz, Vanessa Salete de Paula, Elisangela Ferreira da Silva, Kycia Maria Rodrigues do Ó, Flavio Augusto Pádua Milagres, Marcelo Santos Cruz, Francisco Inácio Bastos, Jurema Corrêa da Mota, Priscila Pollo-Flores, Erotildes Leal, Ana Rita Coimbra Motta-Castro, Lia Laura Lewis-Ximenez, Elisabeth Lampe, Livia Melo Villar

**Affiliations:** 1 Laboratory of Viral Hepatitis, Oswaldo Cruz Institute, FIOCRUZ, Rio de Janeiro, Brazil; 2 Molecular Virology Laboratory, Oswaldo Cruz Institute, FIOCRUZ, Rio de Janeiro, Brazil; 3 São Lucas Hospital, Petropolis, Rio de Janeiro, Brazil; 4 Medicine Faculty, Federal University of Tocantins, Palmas, Brazil; 5 Institute of Psychiatry, Federal University of Rio de Janeiro, Rio de Janeiro, Brazil; 6 Institute of Communication and Scientific Information & Technology for Health, Oswaldo Cruz Foundation, Rio de Janeiro, Brazil; 7 Antonio Pedro University Hospital, Federal Fluminense University, Rio de Janeiro, Brazil; 8 Federal University of Rio de Janeiro, Faculty of Medicine, Rio de Janeiro, Brazil; 9 Federal University of Mato Grosso do Sul and FIOCRUZ-MS, Campo Grande, MS Brazil

**Keywords:** Hepatitis B virus, Oral fluid, Enzyme immunoassay, Prevalence, Diagnosis

## Abstract

**Background:**

Hepatitis B virus (HBV) testing in oral fluid samples may provide advantages in diagnosis, screening or prevalence studies, especially among individuals with venous access difficulties. This study aims to optimize one commercially available assay for detecting total anti-HBc marker in oral fluid samples and to evaluate its utility under real life conditions in different settings for the purposes of prevalence and diagnostic studies.

**Methods:**

Oral fluid was collected using a Salivette device and some parameters were initially evaluated: type of elution buffer and sample volume. Thereafter, the utility of oral fluid samples for detection of anti-HBc was evaluated in real life conditions in which, 1296 individuals gave serum and oral fluid samples. All serum samples were submitted to commercial EIAs to detect total anti-HBc, according to the manufacturer’s instructions and oral fluid samples according to previous optimization.

**Results:**

In optimization evaluation, PBS/BSA 0.5% and 100 μL of oral fluid (volume was two-fold increased compared to serum in EIA) were chosen as transport buffer and sample volume. In the field study, anti-HBc was detected in 211 out of 1296 serum samples giving overall oral fluid sensitivity of 52.6% and specificity of 96%. Concordance was higher in ambulatory setting (67.7) compared to general population (31.8). Mean ± standard deviation values of optical density/cutoff (OD/CO) in serum samples were higher in false-negative oral fluid samples than those seen in true positive samples. Sensitivity was higher in those presenting active infection compared to anti-HBc isolate and past infection. Sensitivity also increased in the ambulatory group when HCV individuals were excluded.

**Conclusions:**

It was possible to optimize a commercial EIA for detecting anti-HBc in oral fluid samples and where the highest concordance was found in ambulatory settings and among individuals with active infection.

## Background

Hepatitis B virus (HBV) represents a substantial health, social and economic burden, with a worldwide estimated of 257 million chronic HBV carriers [1]. In Brazil, 218,257 confirmed cases of HBV were reported from 1999 to 2017. The majority of these cases are concentrated in the Southeast (35.2%), followed by South (31.6%), North (14.3%), Northeast (9.7%) and Midwest (9.2%). The incidence of HBV cases per 100,000 inhabitants in 2017 was 11.3 in North, 2.8 in Northeast, 5.4 in Southeast, 14.3 in South and 6.7 in Midwest regions [2].

Screening of infected, cured and vaccinated individuals is necessary to identify the presence of chronically infected reservoirs, immune and susceptible individuals [3]. Diagnosis of HBV infection is made using serum or plasma samples [4] collected by venipuncture, which is invasive, expensive and potentially painful and arduous for some individuals including drug users, patients under hemodialysis, the obese and the elderly. In regions where financial resources are scarce, it would be beneficial to use methods with low cost and biological risk, such as oral fluid samples. Their collection is less invasive, less painful, simpler and safer than blood collection, allowing collection of a vast number of samples for epidemiological and prevalence studies [5–9].

Oral fluid contains saliva from the salivary glands and gingival crevicular fluid, which is a transudate plasma derived from the capillary bed beneath the tooth–gum margin [10, 11]. The primary drawback of this sample source however, is that the concentration of IgG in oral fluid has been reported to be substantially lower (average 300 times) when compared to its concentration in serum [12–15].

The use of oral fluid samples as a noninvasive alternative to blood for the detection of virus-specific antibodies was first promoted by Parry et al. [16]. Since then these samples have been used to detect Varicella, Herpes simplex, human immunodeficiency virus (HIV), Hepatitis A virus (HAV) and Hepatitis C virus (HCV) markers [11, 15–21]. HBV markers have previously been detected in oral fluid samples, especially the surface antigen of the hepatitis B virus (HBsAg) [5, 7, 22–29]. However, few studies have evaluated the utility of oral fluid samples in detecting antibodies directed against the core protein (anti-HBc marker). In these studies, sensitivities vary from 13.0 to 85.9% and specificities range from 78.0 to 100.0% [9, 30–32].

Anti-HBc appears shortly after HBsAg in acute infection (Anti-HBc IgM) and remains detectable in patients with resolved HBV infections and among chronic cases (anti-HBc IgG) of HBV infection [4]. Since total anti-HBc marker indicates previous contact with the virus, assessment using oral fluid sample could help the surveillance and control of HBV.

This study aims to optimize one commercially available assay for detecting total anti-HBc marker in oral fluid samples and to evaluate its utility under real life conditions in different settings for the purposes of prevalence and diagnostic studies.

## Methods

### Study population

Individuals were recruited at the National Reference Laboratory for Viral Hepatitis (NRLVH) in the Oswaldo Cruz Institute (Rio de Janeiro, Brazil) to give paired serum and oral fluid samples for the optimization of assay conditions. These individuals were recruited in a non-probabilistic method using consecutive sampling and these samples were used only for optimization of the assay conditions.

To evaluate the anti-HBc assay for oral fluid under real life conditions, a total of 1296 individuals were recruited from different serological profiles and different regions.

Serological profiles showed 57 individuals with active infection (HBsAg^+^/anti-HBc^+^/anti-HBs^−^ or HBsAg^+^/anti-HBc^−^/anti-HBs^−^), 37 individuls with anti-HBc isolate (HBsAg−/Anti-HBc+/anti-HBs-), 119 individuals with previous HBV exposure (HBsAg−/anti-HBc+/anti-HBs+), 347 individuals vaccinated for HBV (HBsAg−/ anti-HBc^−^/Anti-HBs^+^) and 736 susceptible individuals (HBsAg^−^/Anti-HBc^−^/Anti-HBs^−^).

The individuals were recruited from different sample collection events as explained below:

Group I (GI) was composed by 291 individuals recruited from the NRLVH ambulatory. The inclusion criteria for this group were acute, chronic or suspected cases of hepatitis B infection and aged more than 18 years. Samples were collected in a non-probabilistic fashion using consecutive sampling.

Group II (GII) was composed by 1005 individuals living in different regions of Brazil. Of these: 441 individuas from Southeast (95 from Macaé and Petrópolis cities, 277 professional beauticians, and 69 crack-cocaine users, all of them residents of Rio de Janeiro state), 336 individuals from North (Tocantins State) and 228 individuals from Midwest (Mato Grosso do Sul State). None of these individuals were recruited in viral hepatitis ambulatory care settings or had been previously diagnosed as HBV infected.

According to the Brazilian Health Ministry, the HBV prevalence rates per 100,000 inhabitants were 2.8 in Rio de Janeiro, 4.6 in Mato Grosso do Sul and 6.3 cases in Tocantins State [2]. In previous reports, incidence of HBsAg in these groups varied from 0.2 to 0.7% and the prevalence of anti-HBc/anti-HBs varied from 9.7 to 12.6% [7, 33, 34]. In Brazil, HBsAg prevalence among crack cocaine users was 6.2% [35], and among beauty professionals prevalence ranged from 0 to 8% [36, 37].

Those recruited from Macaé/RJ, Petrópolis/RJ, Tocantis and Mato Grosso do Sul lived in remote areas and/or deprived communities and reported neither parenteral exposure (i.e. did not inject drugs) nor repeated unprotected sexual intercourse. Recruitment of these individuals was previously described [7, 33, 34].

Beauticians more than 18 years of age were recruited at a fair aiming to promote knowledge, to encourage technical improvement and to stimulate entrepreneurship among beauticians while crack-cocaine users aged 18–24 were recruited when they reported using crack-cocaine on 3 or more days/week in the last 3 months. Further information about recruitment was described previously [35, 37].

A questionnaire comprising demographic (gender and age) and socioeconomic (education level, family income, and home characteristics) status was applied to these individuals to assess associations in the HBV groups evaluated. Data collection took place directly before sample collection.

Samples were collected in a non-probabilistic manner using consecutive sampling. Data concerning the severity of HBV infection in infected participants were unknown at the time of collection. All study participants gave informed consent obtained from the Ethics Committee of Oswaldo Cruz Institute under CAAE number 34055514.9.0000.5248. Each participant (or legal guardian) gave informed consent before entering the study. Laboratory results were sent to participants and, in the case of carriers, they were referred to health services for orientation and treatment.

### Sample collection and laboratory analysis

Blood samples were collected by venipuncture and centrifuged to obtain serum. Oral fluid was obtained using a commercial device (Salivette, Sarstedt, Germany) and processed as previously described [28]. All samples were stored at − 20 °C until analysis.

All serum samples were submitted to commercial Enzyme immunoassays (EIAs) to detect total anti-HBc antibodies directed against HBV surface antigen (anti-HBs) and HBsAg, (ETI-AB-COREK-PLUS, ETI-MAK-4, and ETI-AB-AUK-3, Diasorin, Italy, respectively) according to the manufacturer’s instructions. Reactive samples were retested to confirm these results.

All oral fluid samples were also tested with the EIA ETI-AB-COREK-PLUS (Diasorin, Italy), designed to detect total anti-HBc in serum. The cut off was calculated according to the manufacturer’s instructions for both serum and oral fluid samples. Samples with optical density / cutoff values (OD/CO) above 1.100 were considered non-reactive and those below 0.900 were considered reactive Samples with values between 0.900 and 1.100 were considered indeterminate and retested in duplicate, those that remained undetermined were excluded from the analysis.

Serum samples from the field study were also tested for the presence of antibody against hepatitis C virus (Murex anti-HCV -version 4.0, Diasroin, Italy) and anti-HCV positivity was evaluated in the performance of anti-HBc detection in oral fluid samples.

### Optimization of anti-HBc assay in the panel of oral fluid samples

The first parameter evaluated to optimize EIA using oral fluid to detect anti-HBc was the transport buffer. In this analysis ten paired serum and oral fluid samples were obtained from five anti-HBc reactive individuals and five anti-HBc negative individuals [38]. Five transport buffers were evaluated: (T1) phosphate buffered saline (PBS) pH 7.2; (T2) PBS/Tween 20 0.05%; (T3) PBS/Tween 20 0.05%/ 0.005% sodium azide; (T4) PBS/Tween20 0.2%/ bovine serum albumin (BSA) 5%, and (T5) PBS/BSA 0.5%. These buffers were chosen as they have previously been used to evaluate HBsAg marker in oral fluid [28].

The second parameter was sample volume and in this analysis, 15 anti-HBc reactive and 16 anti-HBc negative individuals were tested. Two volumes were tested: (V1) 100 μL of oral fluid sample + 50 μL of neutralization buffer; (V2) 100 μL of oral fluid sample + 25 μL of neutralization buffer (in sera: 50 μL of sample + 50 μL of neutralization buffer + 50 μL of sample dilution).

All assays were done in duplicate and positive results in serum samples were retested to confirm the results. Only reactive samples were included in this analysis.

### Data analysis

Using anti-HBc detection in serum samples from commercial standard EIA as the benchmark, we cross-compared standard results with actual findings with respect to sensitivity, specificity, and positive and negative predictive values. In addition, ROC (Receiver Operating Characteristics) curves were fitted seeking optimal cut-offs, as explained in the classic paper by Van der Schouw et al. [39].

Descriptive statistics comprise the mean ± the standard deviation, with a preliminary assessment using contingency tables and respective statistics. Categorical variables were compared between groups using the chi-square test or Fisher’s exact test, and continuous variables were analyzed using the Mann–Whitney U test. A *p*-value of < 0.05 was considered significant.

Concordance between the results obtained for the paired oral fluid and sera samples was assessed using the Kappa index (k). According to international standards, findings should be interpreted as follows: < 0.20 corresponds to poor agreement; 0.21–0.40 as fair agreement; 0.41–0.60 as moderate agreement; 0.61–0.80 as good agreement, and 0.81–1.00 corresponds to very good agreement [40].

Bivariate analysis addressed and cross-compared sociodemographic characteristics, stratifying data for groups I, II and II. The serological profile of seromarkers (i.e. HBsAg, anti-HBc and anti-HBs) of the patients was analyzed by subgroup and serological status.

Analyses were performed using GraphPad InStat 3.01 (GraphPad Software, San Diego, CA), MedCalc 9.2.1.0 (MedCalc Software, Mariakerke, Belgium), as well as the Statistical Package for the Social Sciences (SPSS for Windows, release 10.0; SPSS Inc., Chicago, IL).

Each figure combines one scatter plot (“dotplot”), with the respective correlation index (R^2^), as well as the relevant ROC curve (plotting standard and specific D.O., yielding a curve cross-comparing false-positive and true-positive samples). Graphs were fitted using the open source software R 3.5.0, specifically using the ggplot 2 and plotROC libraries.

## Results

### Laboratory parameters evaluation

**Fig. 1:**
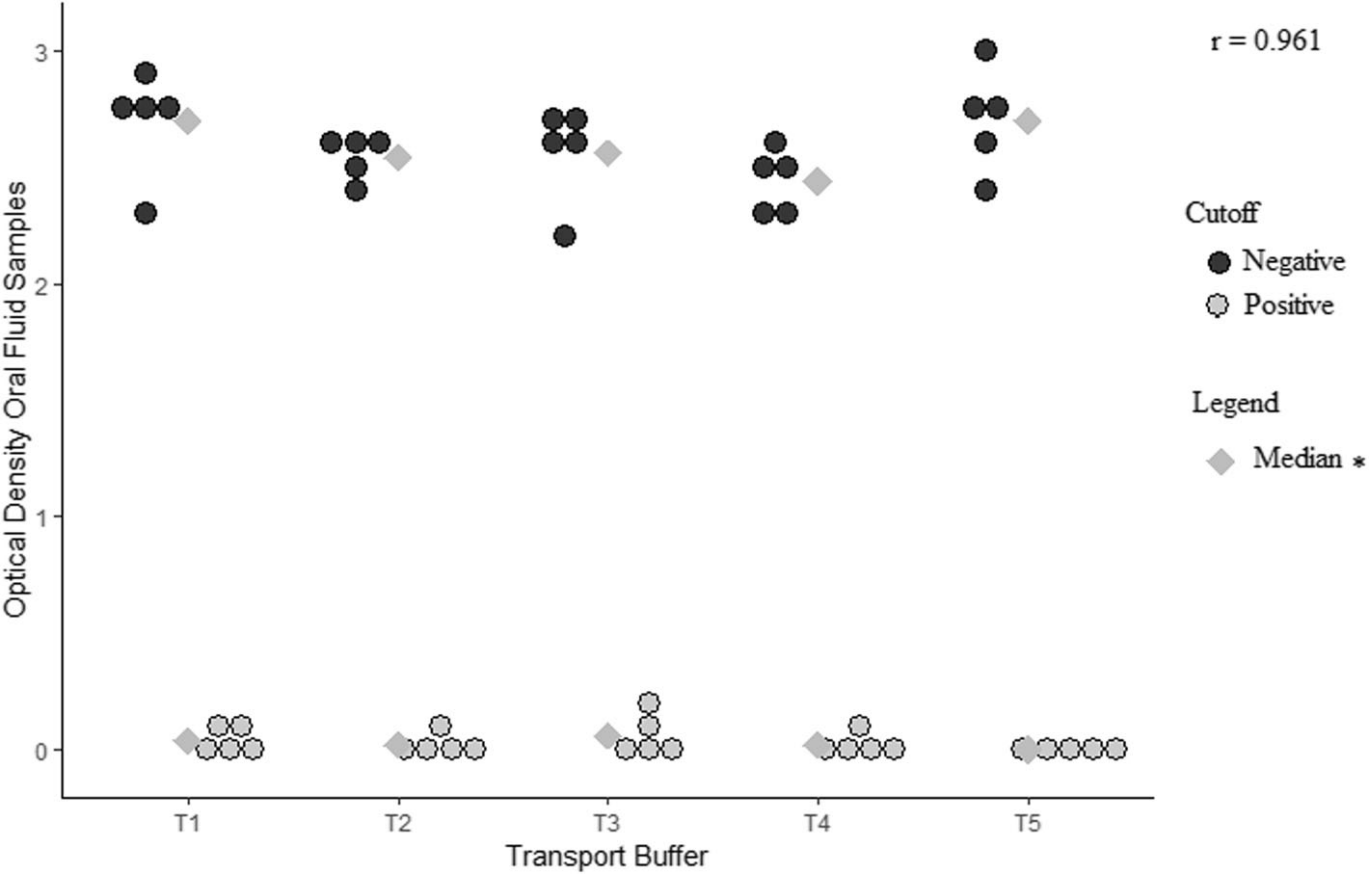
DotPlot Optical Density obtained in oral fluid samples according to transport buffer. Transport Buffers: (1) PBS pH 7.2; (2) PBS/Tween 20 0.05%; (3) PBS/Tween 20 (0.05%)/Sodium azide (0.005%); (4) PBS/Tween 20 (0.2%)/BSA 5%; (5) PBS/BSA 0.5%. Notes: (a) Correlation coefficient Pearson: 0.961; (b) Mean and standard deviation not shown due to the low number of observations among those transfer buffers

**Fig. 2:**
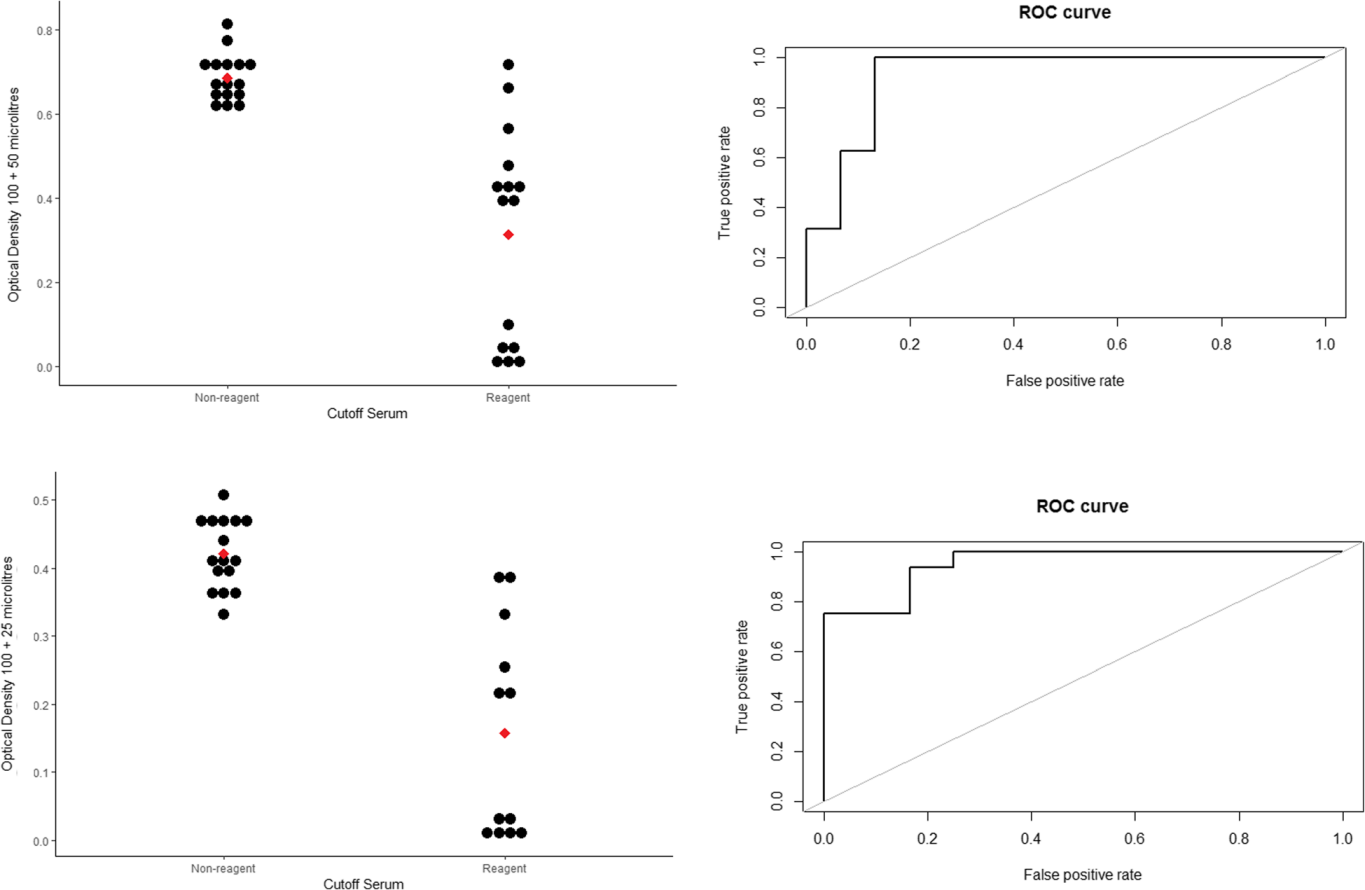
DotPlot Optical Density obtained according to different volumes of oral fluid sample on assay and their respective ROC curves

Transportation buffer and volume of sample in the assay were evaluated to detect total anti-HBc marker using oral fluid samples in a commercial EIA. The transport buffer PBS/BSA 0.5% was chosen as the OD values were closer to the OD values among serum samples (Fig. 1). A 100 μL sample volume of oral fluid and 25 μL of neutralization buffer was chosen by virtue of their lower OD values in positive samples (Fig. 2).

### Field study evaluation

#### Demographic characteristics

The predominant gender among all individuals was female however it was not a significant variable. Mean age ± standard deviation was 50.5 ± 13.4; 35.6 ± 17.5; 36.8 ± 17.8 in GI, GII and all population, respectively. Most individuals were aged less than 40 years (53.3%), had completed high school (29.6%), received a monthly income U$276.00 to 828.00 (32.2%) and did not have hepatitis C virus. There was an association observed between these characteristics and the three groups evaluated (Table 1).

**Table 1:** Socio-demographic characteristics according to each group and HBV serological profile

Data	GI Ambulatory (291) n(%)	GII Regions (1005) n(%)	Active infection (57) n (%)	Anti-HBc isolate (37) n (%)	Previous HBV exposure (119) n (%)	Vaccinated HBV individuals (347) n (%)	Susceptible individuals (736) n (%)	Total (1296) n (%)
Gender								
Female	175 (60.1)	565 (56.2)	39 (68.4)	23 (62.2)	63 (52.9)	195 (56.2)	446 (60.6)	740 (57.1)
Male	108 (37.1)	385 (38.3)	18 (31.6)	13 (35.1)	46 (38.7)	135 (38.9)	263 (35.7)	493 (38.0)
Age (years)								
< 40	63 (21.6)	628 (62.5)	12 (21.0)	22 (59.5)	55 (46.2)	166 (47.8)	387 (52.5)	691 (53.3)
≥ 40	219 (75.3)	295 (29.4)	45 (78.9)	13 (35.1)	47 (39.5)	157 (45.2)	306 (41.6)	514 (39.7)
Mean ± standard deviation	50.5 ± 13.4	35.6 ± 17.5	49.6 ± 14.7	44.3 ± 17,5	36.4 ± 19.8	38.3 ± 17.1	36.9 ± 17.7	36.8 ± 17.8
Education level								
Basic education	64 (22.0)	130 (12.9)	15 (26.3)	7 (18.9)	27 (22.7)	47 (13.5)	98 (13.3)	194 (15.0)
Elementary School	77 (26.5)	125 (12.4)	17 (29.8)	9 (24.3)	19 (16.0)	54 (15.6)	103 (14.0)	202 (15.6)
High school	88 (30.2)	296 (29.4)	16 (28.1)	10 (27.0)	18 (15.1)	105 (30.2)	235 (31.9)	384 (29.6)
Graduate	28 (9.6)	87 (8.6)	4 (7.0)	2 (5.4)	6 (5.4)	35 (10.1)	55 (7.5)	105 (8.8)
Income (according to Brazilian Minimum salary)								
< US$276.00	14 (4.8)	38 (3.8)	3 (5.3)	3 (8.1)	7 (5.9)	12 (3.5)	27 (3.7)	52 (4.0)
U$276.00 to 828.00	152 (52.2)	266 (26.5)	16 (28.1)	16 (43.2)	36 (30.2)	101 (29.1)	249 (33.8)	418 (32.2)
> U$828.00	61 (20.9)	241 (24.0)	9 (15.8)	6 (16.2)	14 (11.8)	108 (31.1)	165 (22.5)	302 (23.3)
Anti-HCV presence								
Reactive	197 (68.4)	18 (1.8)	2 (3.5)	17 (45.9)	22 (18.5)	50 (14.4)	124 (16.8)	215 (16.7)
Non-reactive	91 (31.6)	984 (97.9)	52 (91.2)	20 (54.0)	97 (81.5)	296 (85.3)	610 (82.9)	1075 (83.3)

#### Anti-HBc testing in oral fluid according to HBV marker

Among 1296 individuals, anti-HBc marker was detected in 211 serum samples and undetected in 1085. The serological profiles obtained from individuals collected were: active infection (*n* = 57), anti-HBc isolate (*n* = 37), previous HBV exposure (*n* = 119), vaccinated HBV individuals (*n* = 347) and susceptible individuals (*n* = 736).

Overall anti-HBc sensitivity in oral fluid was 52.6%, but differences were observed according to the group and subgroups under study. High concordance was observed in GI (67.7%) followed by Southeast region of GII (42.9%), North region of GII (39.9%) and MidWest region of GII (30.4%). Specificity values were above 94.2% for all groups and subgroups from GII whereas sensitivities vary from 21.6 to 70.6% (Table 2).

**Table 2:** Quality parameters of anti-HBc detection in oral fluid samples using commercial EIA according to locality of sample collection and serological profile

Profile	TP	FN	TN	FP	Sensitivity% (CI%)	Specificity% (CI%)	PPV% (CI%)	NPV% (CI %)	k (CI)
GI) Ambulatory population (291)	72	30	178	11	70.6 (60.7–79.2)	94.2 (89.8–97.1)	86.7 (77.5–93.2)	85.6 (80.0–90.0)	67.7 (58.5–76.8)
G2) Various Brazilian regions (1005)	39	70	864	32	30.0 (21.2–39.9)	96.4 (95.0–97.5)	48.4 (37.3–59.6)	92.5 (91.6–93.3)	31.8 (19.2–44.3)
MidWest region subgroup (228)	8	29	190	1	21.6 (9.8–38.2)	99.5 (97.1–99.9)	88.9 (51.7–99.7)	86.8 (81.5–90.9)	30.4 (7.1–53.6)
North region subgroup (336)	17	31	279	9	35.4 (22.2–50.5)	96.9 (94.1–98.7)	65.4 (44.3–82.8)	90.0 (86.1–93.1)	39.9 (22.4–57.4)
Southeast region subgroup (441)	14	10	395	22	58.3 (36.6–77.9)	94.7 (92.1–96.7)	38.9 (27.2–51.9)	97.5 (96.1–98.4)	42.9 (23.9–61.9)
Active infection (57)	51	4	1	1	93.1 (83.3–98.1)	50.0 (1.26–98.7)	98.2 (93.1–99.5)	20.0 (4.46–57.2)	25.0 (0–87.9)
Anti-HBc isolate (37)	16	21	–	–	43.2 (27.1–60.5)	–	–	–	–
Previous HBV exposure (119)	44	75	–	–	36.9 (28.3–46.3)	–	–	–	–
Vaccinated HBV individuals (347)	–	–	328	19	–	94.5 (91.6–96.7)	–	–	–
Susceptible individuals (736)	–	–	713	23	–	96.9 (95.3–98.0)	–	–	–
All Individuals (1296)	111	100	1042	43	52.6 (45.6–59.5)	96.0 (94.7–97.1)	72.1 (64.3–79.0)	91.2 (89.4–92.8)	54.6 (47.6–61.6)

Legends: *TP* True positive, *FN* False-negative, *TN* True negative, *FP* False-positive, *PPV* Positive Predictive Value, *NPV* Negative Predictive Value, *k* kappa index, *n* number of samples, *CI* confidence interval, –: not determined

According to the virological profile, higher sensitivity values were observed in individuals with active infections (92.7%) when compared to anti-HBc isolate (43.2%) and past infection (36.9%) (Table 2). Additionally, the presence of HBsAg (active infection) was less observed among false-negative samples (*n* = 4) compared to true-positive samples (*n* = 51) (Table 2).

Additionally, sensitivity was higher in individuals without anti-HCV (55.4%) compared to individuals with the infection (41.5%) but no statistical association was observed (*p* = 0.1196). However, these results were particularly high in GI (90.2% vs 41.0%, respectively) and a statistical association was observed (*p* < 0.001) (Data not shown).

**Fig. 3:**
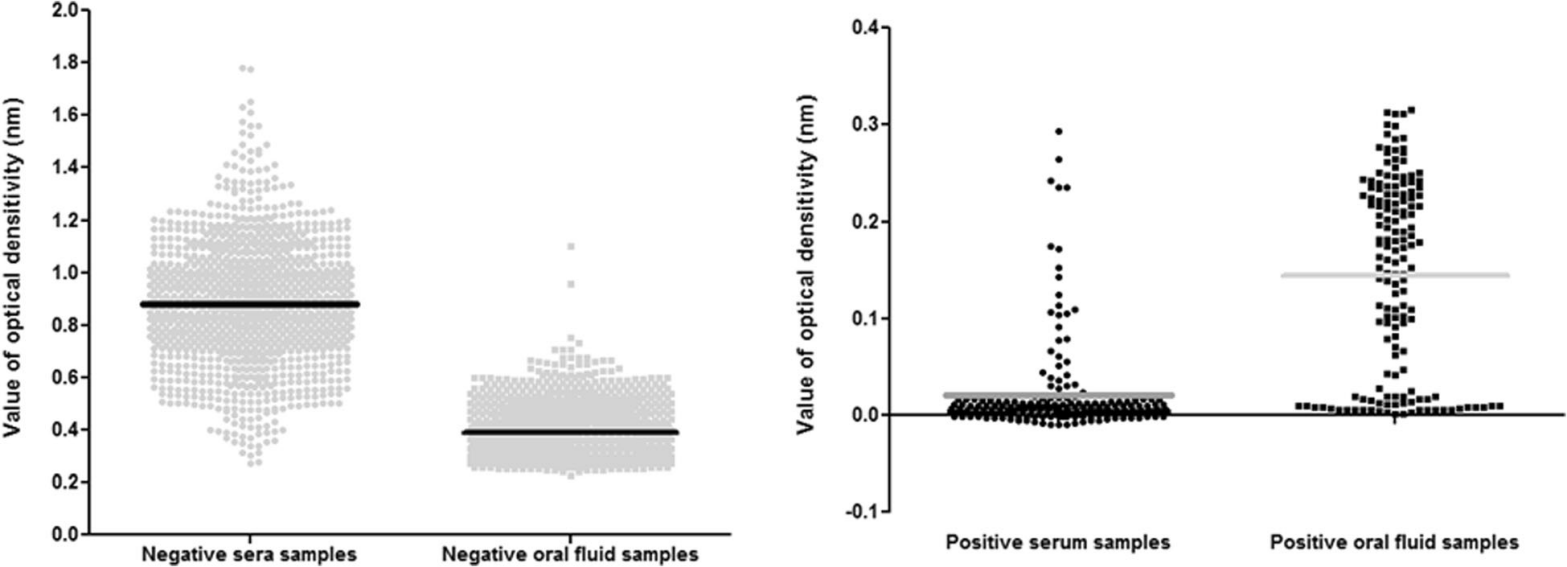
OD ratios of each serum and oral fluid sample plotted according to EIA in negative and positive samples. The y-axis represents the OD ratio. The solid lines represent the average OD ratios for the serum and oral fluid samples, which were 0.881 ± 0.239 and 0.392 ± 0.130 for negative serum and oral fluid samples respectively, and 0.144 ± 0.100 and 0.021 ± 0.048 for positive serum and oral fluid samples respectively

Mean ± standard deviation values of OD/CO in serum samples were calculated between true-positive and false-negative oral fluid samples in each group in order to observe differences between values. Values of OD/CO in serum samples were higher in false-negative oral fluid samples than those seen in true-positive samples, as follows: 1.516 ± 0.251 vs. 0.074 ± 0.333 (*p* < 0.0001) in GI; 0.116 ± 0.219 vs.0.020 ± 0.036 in GII and 1.493 ± 0.473 vs. 0.392 ± 0.338 (*p* < 0.0001) among all individuals from the field study. Negative serum samples showed higher OD values than negative oral fluid samples. Similarly, positive serum samples showed lower OD values than oral fluid samples (Fig. 3).

## Discussion

In this study, a commercial EIA was optimized for anti-HBc detection in oral fluid samples demonstrating good performance in ambulatory group compared to other populations/individuals living in different settings. Primarily, the commercial EIA was adapted for oral fluid samples using elution buffer PBS /BSA 0.5% (buffer 5) - the most appropriate to anti-HBc detection as demonstrated by OD/CO values. This was likely due to the presence of bovine albumin’s minimizing effect upon non-specific reactions. The same buffer has also been used for HBsAg detection in oral fluid samples using optimized commercial EIAs [28]. In addition, the volume of oral fluid sample added to the test was twofold increased in assay compared to serum, probably due to the low amount of antibodies in the former, as seen in similar studies measuring viral hepatitis markers in oral fluid [5, 21, 32, 41].

When anti-HBc assay in oral fluid was evaluated in different groups, good concordance was observed in group I (k = 67.7%) and fair concordance in group II (group, k = 31.8%). These differences could be due to the presence of active infection (acute or chronic cases), since individuals from the ambulatory group tend to have a high probability of presenting serum HBsAg. It is important to note that the oral fluid anti-HBc assay had high sensitivity in individuals presenting active infection compared to those with anti-HBc isolate and past HBV infection. This is in agreement with prior observations of the best HBV assay performance using oral fluid samples from ambulatory settings [31] and among those with active infection with 90.5% sensitivity when only HBV infected individuals were included in the study [9].

The use of the anti-HBc assay with oral fluid samples demonstrated high specificities in all groups/subgroups (over 94.2%). These findings are congruent with studies in different settings, such as viral hepatitis clinics [9, 32], blood donors and injecting drug users [31]. Sensitivities of the anti-HBc assay using oral fluid vary between groups and subgroups; from 21.6% in the Midwest region of Brazil (subgroup from GII) to 70.6% in the ambulatory group (GI), probably due to the high number of active infections in group I. Previous studies also demonstrated low sensitivity of anti-HBc detection in oral fluid samples; 13 and 43% reported by Amado et al. [32] and Nokes et al. [30] respectively. High sensitivity was found among the injecting drug user group (85.9%) [30] which could also be the result of the high number of active infections this group possesses.

Differences in anti-HBc testing performance between the present study and past studies could also be the result of distinct oral fluid collector devices and types of EIA used. In the present study, the Salivette device and Diasorin EIA were used while Nokes et al. used the Oracol collector (Malvern Medical Developments) and Organon Teknika EIA [29]; Fisker et al. used the Omni-SAL® (Saliva Diagnostic Systems, Singapore) and Murex ICE HBc EIA (Murex Biotech Ltd., UK) [30] and Amado et al. used the Orasure® device (Orasure Technologies Inc., Bethlehem, PA, USA) and Organon Teknika EIA [31].

Previous studies have demonstrated the difference of HBV and HCV testing according to oral device [7, 21, 41, 42], but no study has evaluated different devices for anti-HBc testing in oral fluid samples. In the present study, the Salivette device was chosen due to low cost and availability in the Brazilian Market. In addition, this device has been previously used for HBsAg and anti-HCV detection in oral fluid samples with good results [7, 21].

It is interesting to observe that low numbers of false-negative results for oral fluid were found in the Southeast region compared to other regions of Group II. Scalioni et al. [7] have shown that HBsAg titers diminished between 15 and 30 days when stored at 37 °C, the same temperature recorded for several months in the Midwest and North regions of Brazil. However, to our knowledge, no evaluation of the stability of anti-HBc in saliva was performed. The low number of FN in the Southest region samples was probably due to the short interval (at most a few hours) between sample collection and transportation to the laboratory, while in North and Midwest regions, in some cases, the sample interval between collection and transportation to the laboratory was several days (though less than 10 days).

As expected, the OD values were lower in the saliva samples when compared to serum, however, false-negative samples demonstrated in their paired serum samples both low and high OD values, demonstrating that serum anti-HBc concentration was probably not associated with saliva anti-HBc detection. We also observed high sensitivity in the ambulatory group, when HCV subjects were excluded (90.2%), showing the impact of these infections upon the anti-HBc assay using oral fluid samples. Low sensitivity values of the anti-HBc assay were observed in HIV infected individuals, particularly among those receiving antiretroviral treatment [9], where they suggest interference by the presence of HIV or ARV treatment. To our knowledge, the interference of HCV infection in anti-HBc assays using oral fluid samples has not been previously observed.

The present study has some limitations, such as the absence of additional information on clinical history and status and putative therapeutic regimens. There is no information either on HBV DNA and HBsAg titers in HBsAg reactive serum samples or about problems in the procedures actually implemented in the storage and shipping of samples from distant/remote locations to the reference lab.

## Conclusions

In conclusion, it was possible to optimize a commercial EIA for detecting anti-HBc in oral fluid samples where the highest concordance was found in ambulatory settings and among individuals with active infection.

## Data Availability

The datasets generated and/or analysed during the current study are not publicly available to maintain the privacy and confidentiality of the subjects but are available from the corresponding author upon reasonable request.

## Abbreviations

Anti-HBc IgG: Antibodies directed against the core antigen IgG
Anti-HBc IgM: Antibodies directed against the core antigen IgM
Anti-HBc total: Antibodies directed against the core antigen
Anti-HBs: Antibodies directed against hepatitis B surface antigen
BSA: Bovine serum albumin
CI: Confidence interval
CO: Cut-off value
EIA: Enzyme immunoassay
FN: False negative result (negative in DBS and positive in sera)
FP: False positive result (positive in DBS and negative in sera)
HAV: Hepatitis A virus
HBsAg: Surface antigen of the hepatitis B virus
HBV: Hepatitis B virus
HCV: Hepatitis C virus
HIV: human immunodeficiency Vírus
k: Kappa index
LRNHV: National Reference Laboratory for Viral Hepatitis
n: number of samples
NPV: Negative predictive value
OD: Optical density
PBS: phosphate buffer saline
PPV: Positive predictive value
SD: standard deviation
TN: True negative result (negative in both DBS and sera)
TP: True positive result (positive in both DBS and sera)
